# Recurrent Chronic Idiopathic Urticaria Post-hysterectomy and Loss of Xolair Effectiveness: A Case Report

**DOI:** 10.7759/cureus.81624

**Published:** 2025-04-02

**Authors:** Miriam Al-Saedy, Salsabeal Al-Saedy, David Robinson

**Affiliations:** 1 Internal Medicine, Virginia Mason Medical Center, Seattle, USA; 2 Medicine, Elson S. Floyd College of Medicine, Spokane, USA; 3 Allergy and Immunology, Virginia Mason Medical Center, Seattle, USA

**Keywords:** biologic, biologic treatment, chronic idiopathic urticaria, chronic spontaneuous urticaria, hives, post-op recurrence

## Abstract

We present the case of a 44-year-old woman with a history of chronic idiopathic urticaria (CIU) who initially achieved remission with omalizumab (Xolair) but experienced a relapse following a hysterectomy. Despite reinitiating treatment, her symptoms persisted, requiring escalating doses of antihistamines and systemic corticosteroids. This case highlights the potential role of hormonal shifts and surgical stress in triggering urticaria recurrence and contributing to biologic treatment failure, though causality remains unproven due to the lack of objective hormonal or immunologic data. It underscores the importance of closely monitoring treatment response over time and exploring alternative therapeutic strategies when standard approaches prove inadequate. Further research is needed to elucidate the mechanisms underlying post-surgical urticaria exacerbations and optimize long-term management for affected patients.

## Introduction

Chronic idiopathic urticaria (CIU) is characterized by recurrent hives lasting longer than six weeks without an identifiable external trigger [1]. It significantly impacts patients' quality of life, often leading to distress, sleep disturbances, and reliance on long-term pharmacotherapy [1]. Delayed pressure urticaria (DPU) is a physical subtype of CIU, manifesting as swelling and wheals in response to sustained pressure [2]. First-line treatment typically includes second-generation antihistamines, with escalation to omalizumab (Xolair) or immunosuppressants for refractory cases [3]. While biologics like Xolair have revolutionized CIU management, treatment response varies, and cases of secondary treatment failure remain a clinical challenge [3].

Surgical procedures, particularly those affecting hormonal and immune homeostasis, have been implicated in urticaria flares [3,4]. Hormonal fluctuations, surgical stress, and immune modulation can exacerbate urticarial activity, particularly in women undergoing procedures such as hysterectomy [5,6].

The relationship between hormonal changes and CIU is not fully understood, but estrogen and progesterone have been suggested to influence mast cell activity and histamine release. Hormonal influences on mast cell activity and urticaria exacerbation post-gynecologic surgeries have been explored in various studies. Estrogen and progesterone fluctuations are known to modulate mast cell degranulation and histamine release, which could contribute to urticaria recurrence after surgical procedures that alter hormonal balance. For instance, a study highlighted that estrogen enhances mast cell activation and increases vascular permeability, potentially exacerbating allergic and inflammatory conditions, including urticaria [7]. Additionally, research demonstrated a correlation between menstrual cycle phases and urticaria severity, further supporting the role of hormonal modulation in mast cell-driven diseases [8,9].

However, direct hormonal or immune markers were not measured in this case, limiting our ability to establish causality. This report presents a case of a patient with a well-controlled history of CIU who experienced recurrence and Xolair resistance following a hysterectomy, emphasizing the need for further research into the impact of hormonal and surgical factors on chronic urticaria.

## Case presentation

A 44-year-old woman with a history of chronic idiopathic urticaria (CIU) and delayed pressure urticaria (DPU) presented to our outpatient allergy clinic in May 2024 for evaluation of recurrent symptoms. She initially developed CIU in 2019, characterized by spontaneous hives and delayed pressure-induced lesions predominantly affecting the palms and soles. Her condition was refractory to antihistamines and systemic corticosteroids until she was started on omalizumab (Xolair), which led to significant symptom control and eventual remission. By 2022, she successfully tapered off Xolair without a recurrence of symptoms.

In March 2024, following a hysterectomy for abnormal uterine bleeding, the patient experienced a relapse of CIU with symptom severity comparable to her initial presentation. She reported recurrent hives that were steroid-dependent, requiring a daily dose of 10 mg prednisone to maintain symptom control. Antihistamine therapy included cetirizine 10 mg daily and diphenhydramine as needed. She described the lesions as delayed pressure-induced with prominent involvement of the palms and soles, consistent with her prior episodes.

Clinical course

Initial Evaluation

Physical examination during the initial visit revealed no active lesions, but the patient provided photographs documenting her hives, which were consistent with delayed pressure-induced urticaria. Given the history of steroid dependence and minimal relief with antihistamines, her cetirizine dose was increased to 20 mg daily, and hydroxyzine 50 mg at night was reintroduced. It was discussed that Xolair could be pursued if symptoms persisted despite antihistamine optimization [1,3].

Follow-Up Visit

At her follow-up visit, the patient reported persistent hives and continued dependence on prednisone. Despite increased antihistamine dosing, there was minimal improvement in symptom control. The decision was made to reinitiate Xolair therapy at a dose of 300 mg every four weeks. It is important to note that omalizumab typically requires three to six doses to assess efficacy [1,3], although this patient had previously achieved efficacy on Xolair. The patient was informed about the need to receive the first three injections in the clinic before transitioning to home administration [4,5].

Subsequent Management

Despite receiving two doses of Xolair, the patient continued to experience high levels of urticarial activity. Her prednisone dose was increased to 15 mg twice a day, and fexofenadine was added to her regimen as an additional nonsedating antihistamine. A cholinergic component was suspected based on the clinical worsening of symptoms with physical exertion and increased body temperature; however, no diagnostic testing (e.g., exercise challenge, methacholine) was performed to confirm this suspicion [6,10]. 

Her clinical course supported a diagnosis of refractory CIU exacerbated by delayed pressure and suspected cholinergic components [11]. Throughout her clinical course, the patient faced significant morbidity due to persistent symptoms and the need for corticosteroids. The recurrent nature of her disease, particularly following surgical intervention, raised concerns about the potential impact of hormonal changes and immune dysregulation on disease pathogenesis. However, objective markers such as anti-omalizumab antibody testing or serum IgE monitoring were not performed, representing a diagnostic gap. The lack of response to Xolair, despite previous success, highlighted the variability in biologic therapy efficacy and the potential for secondary treatment failure [12,13]. The patient's treatment plan focused on achieving symptom control while minimizing corticosteroid dependence. She continued Xolair therapy with the hope of a delayed response and was monitored closely for the need for further therapeutic adjustments. Follow-up assessments were planned to evaluate the efficacy of the revised treatment regimen and the feasibility of tapering prednisone [14].

## Discussion

This case highlights the complexity of managing CIU, particularly when exacerbated by surgical interventions and refractory to standard therapies. The recurrence of symptoms post-hysterectomy suggests the potential influence of hormonal changes and immune dysregulation on urticaria activity. Estrogen and progesterone fluctuations, along with surgical stress, have been proposed as contributors to mast cell degranulation and increased histamine release, which may explain the patient's symptom recurrence [5]. However, without baseline and post-surgical lab data (e.g., estrogen/progesterone levels, inflammatory markers), the role of hormonal shifts remains speculative. Interestingly, her prior surgical history included a bariatric gastric sleeve surgery, which did not trigger an urticarial flare. Furthermore, there may be a connection between general anesthesia and CIU flares, and further studies assessing how anesthesia plays a role in urticarial flares would be beneficial [15].

While omalizumab remains a cornerstone of treatment for refractory CIU, variability in response is an ongoing clinical challenge. This patient's relapse, despite previously effective therapy, suggests the potential for secondary biologic resistance. Mechanisms such as anti-drug antibodies, receptor downregulation, and altered IgE-mediated pathways may contribute to treatment failure, though these were not directly assessed in this case. Monitoring response to Xolair over time is critical to assess its efficacy and to determine whether adjunctive treatments, such as leukotriene receptor antagonists or cyclosporine, are necessary [6,10]. The ultimate goal in management is to reduce dependence on systemic corticosteroids and optimize antihistamine regimens to improve quality of life and minimize adverse effects [11,12].

Management strategies must be tailored to address both the acute exacerbation and long-term disease control. This includes closely tracking symptom progression, evaluating for potential triggers, and considering alternative pathways for treatment when biologics fail. Further studies are needed to better understand the interplay between hormonal changes and CIU, particularly in the context of surgical interventions, to guide treatment decisions and improve patient outcomes [13,14].

## Conclusions

This case highlights the complexity of managing CIU, particularly when exacerbated by surgical interventions and refractory to standard therapies. The patient’s post-hysterectomy exacerbation of urticaria underscores the need to consider hormonal and immunologic shifts as potential triggers. However, due to the lack of direct hormonal or immune assessments, causality cannot be established. Additionally, the lack of response to omalizumab despite prior success raises questions about factors influencing biologic treatment failure, such as altered immune pathways or changes in disease phenotype. Future studies should consider evaluating predictive markers such as serum tryptase, IgE, and cytokine profiles to improve treatment stratification.

Clinicians managing refractory CIU should adopt a personalized approach, balancing effective symptom control with minimizing adverse effects. Further research is necessary to elucidate the mechanisms of post-surgical urticaria triggers and identify predictive markers for biologic therapy responsiveness. Insights gained may guide more targeted and effective management strategies for patients with complex presentations of CIU.

## References

[1] Kaplan A, Lebwohl M, Giménez-Arnau AM, Hide M, Armstrong AW, Maurer M (2023). Chronic spontaneous urticaria: focus on pathophysiology to unlock treatment advances. Allergy.

[2] Kulthanan K, Ungprasert P, Tuchinda P, Chularojanamontri L, Charoenpipatsin N, Maurer M (2020). Delayed pressure urticaria: a systematic review of treatment options. J Allergy Clin Immunol Pract.

[3] Bousquet J, Humbert M, Gibson PG, Kostikas K, Jaumont X, Pfister P, Nissen F (2021). Real-world effectiveness of omalizumab in severe allergic asthma: a meta-analysis of observational studies. J Allergy Clin Immunol Pract.

[4] Zuberbier T, Peter J, Staubach P, Chularojanamontri L, Kulthanan K (2023). Potential therapeutic approaches for chronic urticaria: beyond H1-antihistamines and biologics. J Allergy Clin Immunol Pract.

[5] Sánchez-Borges M, Ansotegui IJ, Baiardini I (2021). The challenges of chronic urticaria part 1: epidemiology, immunopathogenesis, comorbidities, quality of life, and management. World Allergy Organ J.

[6] Kolkhir P, Church MK, Weller K, Metz M, Schmetzer O, Maurer M (2017). Autoimmune chronic spontaneous urticaria: qhat we know and what we do not know. J Allergy Clin Immunol.

[7] Theoharides TC, Alysandratos KD, Angelidou A (2012). Mast cells and inflammation. Biochim Biophys Acta.

[8] Bernstein JA, Bouillet L, Caballero T, Staevska M (2021). Hormonal effects on urticaria and angioedema conditions. J Allergy Clin Immunol Pract.

[9] Mostmans Y, Maurer M, Richert B (2024). Chronic spontaneous urticaria: evidence of systemic microcirculatory changes. Clin Transl Allergy.

[10] Fok JS, Kolkhir P, Church MK, Maurer M (2021). Predictors of treatment response in chronic spontaneous urticaria. Allergy.

[11] Konstantinou GN, Konstantinou GN (2020). Psychological stress and chronic urticaria: a neuro-immuno-cutaneous crosstalk. A systematic review of the existing evidence. Clin Ther.

[12] Church MK, Kolkhir P, Metz M, Maurer M (2018). The role and relevance of mast cells in urticaria. Immunol Rev.

[13] Zuberbier T, Ensina LF, Giménez-Arnau A (2024). Chronic urticaria: unmet needs, emerging drugs, and new perspectives on personalised treatment. Lancet.

[14] Pathania YS (2024). Treatment options in refractory chronic spontaneous urticaria. Curr Opin Allergy Clin Immunol.

[15] Kim JC, Kim DC, Choi YW, Lee ES, Choi JW (2022). Association of chronic spontaneous urticaria with the first exposure to general anaesthesia. Clin Exp Allergy.

